# Issue Information

**DOI:** 10.1002/cam4.125

**Published:** 2014-06-04

## Aims and Scope

*Cancer Medicine* is a peer-reviewed, open access, interdisciplinary journal providing rapid publication of cutting-edge research from global biomedical researchers across the cancer sciences. The journal considers submissions from all oncologic specialties, including, but not limited to, the following areas:

### Cancer biology

Molecular biology • cellular biology • molecular genetics • genomics • immunology • epigenetics • metabolic studies • proteomics • cytopathology • carcinogenesis • drug discovery and delivery

### Clinical cancer research

Translational research • clinical trials • chemotherapy • radiation therapy • surgical therapy • clinical observations • clinical guidelines • genetic consultation • ethical considerations

### Cancer prevention

Behavioral science • psychosocial studies • screening • nutrition • epidemiology and prevention • community outreach

*Cancer Medicine* publishes original research articles, systematic reviews, meta-analyses, and research methods papers, along with invited editorials and commentaries. Original research papers must report well-conducted research with conclusions supported by the data presented in the paper.

We aim to be a truly global forum for high-quality cancer research, and we think that the best research should be published and made widely accessible as quickly as possible. *Cancer Medicine* publishes papers submitted directly to the journal and those referred from a select group of prestigious journals published by Wiley-Blackwell.

*Cancer Medicine* is a Wiley Open Access journal, one of a new series of peer-reviewed titles publishing quality research with speed and efficiency. For further information visit the Wiley Open Access website at http://www.wileyopenaccess.com.

## Open Access and Copyright

All articles published by *Cancer Medicine* are fully open access: immediately freely available to read, download and share. All articles accepted from 14 August 2012 are published under the terms of the Creative Commons Attribution License. All articles accepted before this date were published under a Creative Commons Attribution Non-Commercial License. The Creative Commons Attribution License permits use, distribution and reproduction in any medium, provided the original work is properly cited and allows the commercial use of published articles. Copyright on any research article in a journal published by *Cancer Medicine* is retained by the author(s). Authors grant Wiley a license to publish the article and identify itself as the original publisher. Authors also grant any third party the right to use the article freely as long as its integrity is maintained and its original authors, citation details and publisher are identifi ed. Further information about open access license and copyright can be found at http://www.wileyopenaccess.com/details/content/12f25db4c87/Copyright--License.html.

## Purchasing Print Reprints

Print reprints of Wiley Open Access articles can be purchased from corporatesales@wiley.com.

## Disclaimer

The Publisher and Editors cannot be held responsible for errors or any consequences arising from the use of information contained in this journal; the views and opinions expressed do not necessarily reflect those of the Publisher and Editors, neither does the publication of advertisements constitute any endorsements by the Publisher and Editors of the products advertised.

Wiley Open Access articles posted to repositories or websites are without warranty from Wiley of any kind, either express or implied, including, but not limited to, warranties of merchantability, fitness for a particular purpose, or non-infringement. To the fullest extent permitted by law Wiley disclaims all liability for any loss or damage arising out of, or in connection with, the use of or inability to use the content.

**Editor-in-Chief**

Qingyi Wei, M.D., Ph.D.

The University of Texas MD Anderson Cancer Center, Houston, USA

**Editorial Board**

**Cancer Biology**

Richard J. Ablin

University of Arizona College of Medicine, Tucson, USA

Rosalind Eeles

Institute of Cancer Research, Sutton, UK

Elizabeth A. Grimm

The University of Texas MD Anderson Cancer Center, Houston, USA

Giseli Klassen

Federal University of Parana, Curitiba, Brazil

Gang Li

University of British Columbia, Vancouver, Canada

Youyong Lu

Beijing Institute for Cancer Research, Beijing, China

Kenneth Offit

Memorial Sloan-Kettering Cancer Center, New York, USA

Athanasios G. Papavassiliou

University of Athens Medical School, Athens, Greece

Kenneth Pienta

University of Michigan, An Arbor, USA

Jack A. Schalken

Radboud University Medical Centre, Nijmegen, Netherlands

Kazu Ushijima

National Cancer Center, Tokyo, Japan

David Vaux

Walter and Eliza Hall Institute of Medical Research, Parkville, Australia

Cun-Yu Wang

UCLA School of Dentistry, Los Angeles, USA

Hongyang Wang

Eastern Hepatobiliary Surgery Institute / Hospital, Shanghai, China

Momiao Xiong

The University of Texas Health Science Center, Houston, USA

Li Zhang

The University of Texas MD Anderson Cancer Center, Houston, USA

**Clinical Cancer Research**

Yung-Jue Bang

Seoul National University College of Medicine, Seoul, Korea

Anthony Chan

The Chinese University of Hong Kong, China

Peter J. Fuller

Prince Henry Institute, Victoria, Australia

Maura L. Gillison

Ohio State University Comprehensive Cancer Center, Columbus, USA

Guido Kroemer

University of Paris Descartes, Paris, France

Lalit Kumar

All India Institute of Medical Sciences, New Delhi, India

Razelle Kurzrock

The University of Texas MD Anderson Cancer Center, Houston, USA

Geoffrey Liu

Princess Margaret Hospital, Toronto, Canada

Jiade J. Lu

National University of Singapore, Singapore

Li Mao

University of Maryland, Baltimore, USA

Akira Nakagawara

Chiba University, Chiba, Japan

Pamela L. Paris

University of California, San Francisco, USA

Dimitrios H. Roukos

Ioannina University School of Medicine, Ioannina, Greece

Frank A. Sinicrope

Mayo clinic, Rochester, USA

Jun Wang

BGI-Shenzhen, Shenzhen, China

Glen J. Weiss

Translational Genomics Research Institute, Phoenix, USA

Eric T. Wong

Beth Israel Deaconess Medical Center, Boston, USA

**Cancer Prevention**

Marianne Berwick

University of New Mexico, Albuquerque, USA

Paolo Boffetta

The Tisch Cancer Institute, New York, USA

Eduardo Cazap

Latin-American & Caribbean

Society of Medical Oncology, Buenos Aires, Argentina

Brock C. Christensen

Dartmouth Medical School, Hanover, USA

Steve Dubinett

David Geffen School of Medicine at UCLA, Los Angeles, USA

Kari Hemminki

German Cancer Research Center, Heidelberg, Germany

Zhibin Hu

Nanjing Medical University, Nanjing, China

Stephen D. Hursting

The University of Texas, Austin, USA

Daehee Kang

Seoul National University College of Medicine, Seoul, Republic of Korea

Lambertus A. Kiemeney

Radboud University Medical Centre, Nijmegen, Netherlands

Dongxin Lin

Chinese Academy of Medical Sciences, Beijing, China

Somdat Mahabir

National Cancer Institute, Bethesda, USA

Girish Maru

ACTREC Tata Memorial Centre, Navi Mumbai, India

Andrew Olshan

University of North Carolina, Chapel Hill, USA

Jong Y. Park

H. Lee Moffi tt Cancer Center & Research Institute, Tampa, USA

Susan K. Peterson

The University of Texas MD

Anderson Cancer Center, Houston, USA

Thomas E. Rohan

Albert Einstein College of Medicine, Bronx, USA

Xifeng Wu

The University of Texas MD Anderson Cancer Center, Houston, USA

**Editorial Advisory Board**

Lawrence Baker

University of Michigan, Ann Arbor, USA

David Christiani

Harvard School of Public Health, Boston, USA

Robert B. Diasio

Mayo Clinic, Rochester, USA

B. Mark Evers

Lucille P. Markey Cancer Center, Lexington, USA

Waun Ki Hong

The Univeristy of Texas MD Anderson Cancer Center, Houston, USA

Ernest Hawk

The University of Texas MD Anderson Cancer Center, Houston, USA

Leroy Hood

Institute for Systems Biology, Seattle, USA

James Mulé

H. Lee Moffi tt Cancer Center & Research Institute, Tampa, USA

Philip Agop Philip

Wayne State University, Detroit, USA

Nicholas Vogelzang

Comprehensive Cancer Centers of Nevada, Vegas, USA

